# Editorial: Dynamics of greenhouse gases in forest systems

**DOI:** 10.3389/fpls.2025.1698093

**Published:** 2025-09-30

**Authors:** Julio Javier Diez Casero, Bruno Britto Lisboa, Luciano Kayser Vargas

**Affiliations:** ^1^ University of Valladolid, Valladolid, Spain; ^2^ Department of Agricultural Research and Diagnosis, Secretariat of Agriculture, Livestock, Sustainable Production and Irrigation o Rio Grande do Sul, Porto Alegre, Brazil

**Keywords:** climate change, ecosystem responses, silvicultural approaches, carbon balance, climate regulations

Forest ecosystems play a pivotal role in global climate regulation through their complex interactions with atmospheric greenhouse gases (GHGs), particularly CO_2_, CH_4_, and N_2_O. As the world grapples with the escalating climate crisis, understanding the dynamics of greenhouse gas exchanges in forest systems has become increasingly critical for developing effective climate mitigation and adaptation strategies. This Research Topic presents a comprehensive examination of how various forest systems—both natural and managed—influence greenhouse gas emissions and carbon storage, offering valuable insights into the intricate relationships within the soil-plant-atmosphere continuum.

## The critical role of forests in climate regulation

The urgency of addressing greenhouse gas dynamics in forest systems cannot be overstated. Agriculture and forestry sectors contribute significantly to global greenhouse gas increases, with land-use changes alone accounting for approximately 10-12% of annual anthropogenic emissions (Moore et al., 2018). The extensive replacement of native vegetation with monocultures and pastures has not only enhanced biodiversity loss but also weakened the carbon sink capacity of natural ecosystems. When forest ecosystems succumb to agricultural expansion, they release vast amounts of stored carbon into the atmosphere, fundamentally altering their role from carbon sinks to carbon sources.

However, forests also represent one of our most powerful natural climate solutions. Through photosynthesis, forest ecosystems capture atmospheric CO_2_ and store it in biomass and soils, making them essential carbon sinks (Nzabarinda et al., 2025). The challenge lies in optimizing forest management practices to maximize this carbon sequestration potential while minimizing greenhouse gas emissions, particularly the more potent gases like CH_4_ and N_2_O.

## Research contributions and key findings

The articles compiled in this Research Topic address several critical knowledge gaps in our understanding of forest greenhouse gas dynamics. The research encompasses comparative studies of carbon dynamics under different forest management systems, detailed examinations of greenhouse gas fluxes in natural versus planted forests, and assessments of carbon sequestration capacities across various forest types.

One of the key themes emerging from the contributing articles is the importance of understanding the full spectrum of greenhouse gas exchanges in forest systems. While much attention has historically focused on CO_2_ dynamics, the research presented here highlights the significance of CH_4_ and N_2_O fluxes, which, despite their lower concentrations, have substantially higher global warming potentials. The studies reveal that forest soils can act as both sources and sinks for these trace gases, depending on factors such as soil moisture, temperature, nitrogen availability, and forest management practices.

The Research Topic also illuminates the complexity of carbon balance calculations in forest ecosystems. Traditional approaches that focus solely on above-ground biomass often underestimate the complete carbon picture. The contributing research emphasizes the critical importance of soil organic carbon, which can represent the largest carbon pool in many forest ecosystems. Understanding the factors that influence soil carbon stability and turnover rates is essential for accurate carbon accounting and effective forest management.

## Management implications and future directions

The findings presented in this Research Topic have significant implications for forest management and climate policy. The research demonstrates that sustainable forest management practices can substantially enhance carbon sequestration while reducing greenhouse gas emissions. These practices include selective harvesting techniques (São José et al.), mixed-species plantations (Vallejos-Torres et al.), conservation of old-growth forests (Gu et al.), and the implementation of reduced-impact logging methods.

Particularly noteworthy are the studies examining the effects of different silvicultural approaches on greenhouse gas dynamics. The research shows that management decisions—from species selection to harvest timing—can dramatically influence the net climate impact of forest systems. For instance, certain native tree species demonstrate superior carbon sequestration rates compared to exotic monocultures, while also supporting greater biodiversity and ecosystem resilience.

The Research Topic also addresses the temporal dimensions of forest greenhouse gas dynamics. Long-term studies reveal that the carbon sequestration potential of forests varies significantly over time, influenced by factors such as stand age, disturbance history, and climate variability. This temporal complexity underscores the need for adaptive management approaches that account for changing environmental conditions and forest development stages.

## Technological advances and methodological innovations

Several articles in this Research Topic showcase innovative approaches to measuring and monitoring greenhouse gas fluxes in forest ecosystems. Advanced techniques such as eddy covariance measurements (Xu et al.), chamber-based studies (Guo et al.), and remote sensing applications (Xie et al.) provide unprecedented insights into the spatial and temporal variability of forest-atmosphere gas exchanges. These technological advances are crucial for scaling up local observations to regional and global estimates of forest carbon balance.

The integration of process-based models with empirical observations represents another significant advancement highlighted in this Research Topic. These models help researchers understand the mechanistic drivers of greenhouse gas dynamics and provide tools for predicting future changes under different climate and management scenarios.

## Climate change interactions and ecosystem responses

The research presented here also examines how climate change itself affects forest greenhouse gas dynamics, creating complex feedback loops between forest ecosystems and the global climate system. Rising temperatures, altered precipitation patterns, and increased frequency of extreme weather events all influence forest carbon cycling and greenhouse gas fluxes. Understanding these interactions is crucial for predicting how forests will respond to future climate conditions and for developing climate-resilient forest management strategies.

## Conclusions and future research needs

This Research Topic advances our understanding of greenhouse gas dynamics in forest systems and highlights the critical role that forests can play in climate change mitigation. The research demonstrates that with appropriate management, forest ecosystems can serve as significant carbon sinks while providing numerous co-benefits including biodiversity conservation, watershed protection, and sustainable resource provision.

However, the complexity of forest-atmosphere interactions revealed in these studies also underscores the need for continued research. Future investigations should focus on long-term monitoring programs that capture the full range of environmental variability, mechanistic studies of trace gas production and consumption processes, and the development of management strategies that optimize climate benefits while maintaining ecosystem services.

The path forward requires interdisciplinary collaboration among forest ecologists, atmospheric scientists, land managers, and policymakers. Only through such integrated approaches can we fully realize the potential of forest systems to contribute to global climate stability while meeting society’s diverse needs for forest products and services. The research presented in this Research Topic provides a solid foundation for these efforts and points toward promising directions for future investigation and application.
